# Elective and emergency laparoscopic cholecystectomy in the elderly: early or delayed approach

**DOI:** 10.1186/1471-2318-11-S1-A14

**Published:** 2011-08-24

**Authors:** A Ferrarese, V Martino, M Nano

**Affiliations:** 1 Department of Clinical and Biologic Science, School of Medicine and Surgery “S. Luigi Gonzaga”, AOU San Luigi Gonzaga, Orbassano, Turin - SCDU General Surgery, University of Turin, Italy

## Background

Comparing outcomes of the first Division of Abdominal Surgery of the Saint Louis Hospital of Orbassano (Turin) with the literature, regarding timing and technique of early or delayed laparoscopic cholecystectomy in the management of acute cholecystitis in elderly patients.

## Materials and methods

From January 2005 to December 2009 114 laparoscopic cholecystectomy in the elderly were performed in our surgical division: 67 for gallbladder stones and 47 for acute cholecystitis.

The diagnosis of cholecystitis and gallbladder stones was based on general condition, physical examination, laboratory, radiological findings and sepsis score. For the study we’ve also considered: total hospital stay, timing after and before the operation, kind and duration of operation, conversion to the open procedure, drain and final pathological results.

From this study 29 patients were excluded (17 for choledocolytiasis associated and 12 for hospitalisation > 20 days). We hadn’t excluded patients ASA III and ASA IV: in these patients (27.4 %, 17 ASA III and 4 ASA IV) abdominal pressure not superior of 10 mmHg was used [1]_._

Elderly patients included in the study were 85 (49 M, 36 F). Ordinary Cholecystectomy were peformed in 45 cases and Emergency Cholecystectomy in 40 cases. This last group was further divided into two groups [2-4]: DEA Early, E-DLC, (31 patients operated on within 72 hours from onset of symptoms) and DEA Delayed, D-DLC, (9 patients operated on after 72 hours to 9 days from onset of symptoms).

We’ve also considered the operating team (Table 1) that performed the operation because the first operator’s experience was considered as an important factor in order to evaluate our results [5-11].

**Table 1:** Definitions of equipes.

Team 1	More than 100 laparoscopic cholecistectomy and more than 100 other laparoscopic operations.
Team 2	Less than 100 laparoscopic cholecistectomy and less than 100 other laparoscopic operations.
Team 3	Surgeons in learning curve progression or Resident with expert Surgeon supervisor

## Results

The comparison between elective and emergency operations showed that drain placement and post operation hospital stay were found statistically significant in the emergency group (Table 2). There weren’t any differences regarding team evaluation (Table 3). Concerning the analysis of the E-DLC and D-DLC groups there aren’t any statistical differences (Table 4).

**Table 2:** Ord/DEA.

	OC	DC	P Value
Operation time (min)	75,5 (40-220)	90 (28-200)	0.1874
PO hospital stay (days)	2 (1-10)	3 (2-12)	0.002313
Conversion rate	6.7%	2%	0.3869
Complications	8.5%	2%	0.2352
Drains	16.7%	51%	0.0003
Associated operations	13.3%	12.8%	0.998
Cancer	3%	0	-

**Table 3:** Equipes.

Variabile	E1-E2	E1-E3	E2-E3
Operation time (min)	0.6936	0.6089	0.2759
PO hospital stay (days)	0.3159	0.02131	0.09583
Total hospital stay	0.9362	0.004337	0.004981
Conversion rate	0.1553	0.6677	0.3896
Complications	0.3823	0.998	0.998

**Table 4:** E-DLC/D-DLC.

Parameter	ELC	DLC	P Value
WBC	11.05 (3.73-28.8)	9.05 (2.23-15.6)	0.03264
PCR	1.39 (0.04-45)	0.66 (0.08-23.23)	0.1672
Temperature	14%	2 (7%)	0.5281
Thickened wall	57.4%	13 (48%)	0.4
Pericholecystic fluid	17%	2 (7.4%)	0.25
Distended gallbladder	43.4%	12 (44.4%)	0.998
Operation time (min)	90 (36-330)	85 (28-195)	0.1554
PO hospital stay (days)	3 (2-15)	3 (2-8)	0.6551
Total hospital stay	4 (2-16)	10 (4-16)	p<0,01
Tasso di conversione	5%	0%	0.59
Complications	5%	0%	0.59
Drains	36%	26%	0.3752
Operations associated	8%	15%	0.2353
Cancer	1.6%	0%	0.998

## Conclusions

In contrast with other authors [12,13], laparoscopic cholecystectomy in our elderly patients, when performed with an adequate technique, represents a safe procedure to treat all cases of acute cholecystitis in an emergency setting [14-22]. Our technique represents a standardized surgical strategy to approach acute cholecystitis and cholelytiasis in the elderly in a safe, effective and reproducible manner.
